# Typhoidal Salmonellae: Use of Multi-Locus Sequence Typing to Determine Population Structure

**DOI:** 10.1371/journal.pone.0162530

**Published:** 2016-09-12

**Authors:** Priyanka Sharma, Sushila Dahiya, Veeraraghavan Balaji, Anil Kanga, Preetilata Panda, Rashna Das, Anbumani Dhanraju, Deepak Kumar Mendiratta, Seema Sood, Bimal Kumar Das, Arti Kapil

**Affiliations:** 1 All India Institute of Medical Sciences, New Delhi, India; 2 Christian Medical College, Vellore, India; 3 Indira Gandhi Medical College, Shimla, India; 4 Maharaja Krishna Chandra Gajapati Medical College, Orissa, India; 5 North Eastern Indira Gandhi Regional Institute of Health and Medical Sciences, Shillong, India; 6 Sri Ramachandra Medical College and Research Institute, Chennai, India; 7 Mahatma Gandhi Institute of Medical Sciences, Wardha, India; Public Health England, UNITED KINGDOM

## Abstract

Enteric fever is an invasive infection predominantly caused by *Salmonella enterica* serovars Typhi and Paratyphi A. The pathogens have evolved from other nontyphoidal salmonellaeto become invasive and host restricted. Emergence of antimicrobial resistance in typhoidal salmonellae in some countries is a major therapeutic concern as the travelers returning from endemic countries carry resistant strains to non endemic areas. In order to understand the epidemiology and to design disease control strategies molecular typing of the pathogen is very important. We performed Multilocus Sequence Typing (MLST) of 251 *S*. Typhi and 18 *S*. Paratyphi strains isolated from enteric fever patients from seven centers across India during 2010-2013to determine the population structure and prevalence of MLST sequence types in India. MLST analysis revealed the presence of five sequence types (STs) of typhoidal salmonellae in India namely ST1, ST2 and ST3 for *S*. Typhi and ST85 and ST129 for *S*. Paratyphi A.*S*. Typhi strains showed monophyletic lineage and clustered in to 3 Sequence Types—ST1, ST2 and ST3 and *S*. Paratyphi A isolates segregated in two sequence types ST85 and ST129 respectively. No association was found between antimicrobial susceptibility and sequence types. This study found ST1 as the most prevalent sequence type of *S*. Typhi in India followed by ST2, which is in concordance with previous studies and MLST database. In addition a rare sequence type ST3 has been found which is reported for the first time from the Indian subcontinent. Amongst *S*. Paratyphi A, the most common sequence type is ST129 as also reported from other parts of world. This distribution and prevalence suggest the common spread of the sequence types across the globe and these findings can help in understanding the disease distribution.

## Introduction

Enteric fever remains an important public health problem in developing countries. There are approximately 22 million typhoid cases and 2,16,510 deaths occur per year globally with majority of cases occurring in Asia, especially in the Indian subcontinent [1,2]. The disease is an invasive infection commonly caused by *Salmonella enterica* serovars Typhi and Paratyphi A (hereafter, *S*. Typhi and *S*. Paratyphi A). Despite availability of vaccine and antibiotics effective against typhoidal salmonellae, the disease remains a public health problem in many parts of the developing countries. Also it is increasingly being reported as travel associated infection in non endemic regions. The common treatment options used for enteric fever are ciprofloxacin, ceftriaxone and cefixime [3]. The emergence of quinolone resistant strains in the Indian subcontinent pose a major therapeutic challenge [4,5]. Also there are reports of ceftriaxone resistance from some countries [6,7]. There is a need to undertake studies on the characterization of the strains to understand the epidemiology of disease infections and to design control strategies.

A variety of typing methods are currently in use that vary in reproducibility, reliability and discriminatory power [8]. Most commonly used methods are Multilocus Enzyme Electrophoresis (MLEE), Restriction Fragment Length Polymorphism (RFLP), Pulsed Field Gel Electrophoresis (PFGE), Ribotyping, Multilocus Sequence Typing (MLST) and Whole Genome sequencing (WGS) [9]. With the development of next generation sequencing procedure, sequence based typing methods are gaining more importance due to their reproducibility and high discriminatory power [10].

MLST is a typing method based on the sequencing of housekeeping genes and characterizes isolates on the basis of variation in nucleotide sequences of each locus of the selected genes. The different sequence at each locus are assigned with specific allele numbers and each unique combination of alleles, often called as allelic profile is assigned a sequence type (ST) which is the unambiguous descriptor of the strain [11]. MLST has advantage in long-term global studies and for the assessment of evolutionary relationship among strains. MLST database has been generated by community efforts by submitting the data publically on the website [12] and is a valuable resource for long term epidemiology and population genetics analysis of pathogenic organisms [13, 14].

The calculated age of *S*. Typhi is 15000–50000 years [15] and it is presumed to be evolved from non- typhoidal salmonellae by deletion and acquisition of some genes which makes it a strict human pathogen and causative agent for invasive diseases. A sequence based typing method involving conserved genes might be useful for such a pathogen of relatively recent origin in following the spread and evolution of the pathogen [15].

MLST scheme for *Salmonella enterica* was first described by Kidgel et al [15]. At present there are 9 sequence types for *S*. Typhi and 7 for *S*. Paratyphi A have been documented in the MLST database [12]. An earlier study from India reported the presence of two sequence types in *S*. Typhi from a single centre in north India, in a small number of isolates [16].

The present study was carried out on a wider representation of *S*. Typhi and *S*. Paratyphi A strains to find the evolutionary history and population structure by MLST from different regions of India.

## Material and Methods

The study was conducted in collaboration with 7 participating centers from different parts of India expecting representation of *S*. Typhi strains from different regions of the country.

### Ethics Statement

The study was approved by institutional ethics committees of all participating institutes and subsequently by the co-ordinating center AIIMS.

### Bacterial Isolates

During the study period (March 2010 to March 2013), blood samples were collected from all the patients who presented with enteric fever to the participating hospitals, inoculated in brain heart infusion broth (BD Difco, USA) and incubated at 37°C. Subcultures were made onto MacConkey agar and 5% sheep blood agar (bioMérieux, France). All non lactose fermenting gram negative colonies were identified by standard biochemical tests namely—catalase, motility, sugar fermentation (glucose, sucrose, lactose, mannitol and dulcitol), indole, methyl red voges proskauer, decarboxylase, citrate, malonate, urease and triple sugar iron [17]. The identification was further confirmed by slide agglutination test using specific antisera (Statens Serum Institute, Copenhagen) [18]. *S*. Typhi serovar was characterized by antigenic formula 9,12,Vi:d and *S*. Paratyphi A was characterized by antigenic formula 1,2,12:a.

### Antimicrobial Susceptibility Testing

Antimicrobial susceptibility was determined as per CLSI 2015 [19] using antibiotic disks of chloramphenicol (30 μg), ampicillin (10 μg),, co-trimoxazole (1.25/23.75 μg),, nalidixic acid (30 μg), ciprofloxacin (5 μg), cefixime (30 μg) and ceftriaxone (30 μg) (Himedia Laboratories Ltd, Mumbai, India).Mueller hinton agar medium (BD Difco, Sparks, MD, USA) was inoculated with bacterial inoculum matching to 0.5 McFarland standard and incubated for 16–18 hours at 37°C for antibiotic susceptibility determination. Minimum inhibitory concentration (MIC) for ciprofloxacin and ceftriaxone was determined by E-Test strips (bioMérieux, France) according to manufacturer’s instructions. *E*.*coli* ATCC 25922 was used as a quality control strain for disk diffusion and MIC determination.

### Multilocus Sequence Typing

MLST was carried out in all *S*. Typhi strains submitted by centres who submitted less than 30 strains while for Vellore and New Delhi from where >260 isolates were available, a representation of 90 isolates from each center were taken (30 first consecutive isolates from each year). All *S*. Paratyphi A isolates were characterized by MLST. A total of 251 *S*. Typhi and 18 *S*. Paratyphi A strains were subjected to MLST.

Segments of seven housekeeping genes; *thrA* (aspartokinase+homoserine dehydrogenase), *purE* (phosphoribosylaminoimidazole carboxylase), *sucA* (alpha ketoglutarate dehydrogenase), *hisD* (histidinol dehydrogenase), *aroC* (chorismate synthase), *hemD* (uroporphyrinogen III cosynthase), and *dnaN* (DNA polymerase III beta subunit) were amplified and sequenced for MLST analysis.

Chromosomal DNA was isolated from freshly grown strains using Qiamp commercial DNA isolation kit (*QIAamp* DNA minikit; *Qiagen*, Hilden, Germany). PCR amplification was done for seven housekeeping genes using previously defined primers taken from MLST database as already reported [15]. *S*. Typhi standard strain Ty-2 was used as a positive control and PCR conditions were used as previously described method [16]. PCR product was confirmed by specific bands in Gel electrophoresis.

Sequencing was carried out by the dideoxynucleotide chain termination method, using an automated DNA sequencer ABI PRISM^®^ 310 Genetic Analyzer (Applied Biosystems, USA) using AmpliTaq Gold DNA polymerase (Applied Biosystems, USA). Forward and reverse DNA sequences were aligned and analyzed for each gene fragment in Genedoc v 2.7 [20].

Allele numbers and sequence types were assigned by comparing sequences with public MLST profile database [12].

### Phylogenetic Analysis

The end to end sequences were joined and concatenated sequences of 3666 base pairs were formed for all the isolates. Multiple alignments were done with ClustalX 1.8114 and *S*. Agona, *S*. Paratyphi A, *S*. Newport, *S*. Enteritidis, *S*. Typhimurium and *S*. Heidelberg were taken as outgroups to generate a phylogenetic tree using the Maximum Likelihood method by using MEGA6 v6.1 [21,22]. The evolutionary history was inferred by using the Maximum Likelihood method based on the Tamura-Nei model [21]. Initial tree(s) for the heuristic search were obtained automatically by applying Neighbor-Join and BioNJ algorithms to a matrix of pairwise distances estimated using the Maximum Composite Likelihood (MCL) approach, and then selecting the topology with superior log likelihood value. Codon positions included were 1st+2nd+3rd+Noncoding. All positions containing gaps and missing data were eliminated.

MLST dataset was analysed using eBURST algorithm to find out the evolutionary distance between sequence type variants. The sequence types having pairwise identity to six out of the seven gene fragments belong to same eBURST group as described by by Feil et al. [23]. Analysis was done for all available of *S*. Typhi and *S*. Paratyphi A sequence types in online MLST database to find out evolutionary relationship between sequence types.

Concatenated sequences for all genes were analyzed to find out mutation and recombination for *S*. Typhi and *S*. Paratyphi A sequence types separately using ClonalFrame for 10 runs of 50% consensus each with 100,000 iterations following a burning phase of 100,000 iterations [24,25].

### Analysis of Geographical Distribution of Sequence Types

The global distributions of all the sequence types of *S*. Typhi and *S*. Paratyphi A submitted to MLST database were retrieved from MLST website [12] and were used for the analysis of global distribution of different sequence types and the analysis was represented on a world map [26].

## Results

### Bacterial Isolates

A total of 610 strains were received from all participating centers from the cases of enteric fever, out of which 592 were *S*. Typhi and 18 were *S*. Paratyphi A (Tables 1 and 2).

**Table 1:** Detailed antibiogram of *S*. Typhi strains received from different centers.

Center Name(Total no. of strains)	CHLNo. of Susceptible strains (%)	AMOXNo. of Susceptible strains (%)	COTNo. of Susceptible strains (%)	NANo. of Susceptible strains (%)	CIPNo. of Susceptible strains (%)	CFXNo. of Susceptible strains (%)	CTXNo. of Susceptible strains (%)
Chennai(n = 7)	7(100)	7(100)	7(100)	3(42.8)	3(42.8)	7(100)	7(100)
Orissa(n = 15)	15(100)	14(93.33)	14(93.33)	5(33.4)	5(33.4)	15(100)	15(100)
Shillong(n = 15)	15(100)	15(100)	15(100)	5(33.4)	5(33.4)	15(100)	15(100)
Shimla(n = 20)	20(100)	20(100)	20(100)	7(35.0)	7(35.0)	20(100)	20(100)
Wardha(n = 5)	5(100)	5(100)	5(100)	2(40.0)	2(40.0)	5(100)	5(100)
Vellore(n = 267)	255(95.5)	250(93.4)	252(94.4)	8(32.5)	8(32.5)	267(100)	267(100)
New Delhi(n = 263)	260(98.8)	251(95.4)	260(98.8)	98(37.3)	98(37.3)	263(100)	263(100)
Total (n = 592)	577(97.5)	562 (94.9)	573(96.8)	128(21.6)	128(21.6)	592(100)	592(100)

Abbreviations: CHL (Chloramphenicol), Amox (Amoxicillin) COT (Co-trimoxazole), NA (Nalidixic acid) CIP (Ciprofloxacin), CFX (Cefixime), CTX (Ceftriaxone).

Zone diameters as per CLSI 2015: Susceptible/Resistant–CHL ≥18mm/≤17mm, AMOX ≥23mm/≤22mm, COT ≥16mm/≤16mm, CIP ≥31mm/≤30mm, CFX ≥19mm/≤18mm, CTX ≥23mm/≤22mm [14] (intermediate zone diameters are considered as resistant).

**Table 2:** Detailed antibiogram of *S* Paratyphi A strains received from different centers.

Center Name(Total no. of strains	CHL No. of Susceptible strains (%)	AMOX No. of Susceptible strains (%)	COT No. of Susceptible strains (%)	NANo. of Susceptible strains (%)	CIP No. of Susceptible strains (%)	CFX No. of Susceptible strains (%)	CTX No. of Susceptible strains (%)
Chennai(n = 1)	1(100)	1(100)	1(100)	0	0	1(100)	0(100)
Orissa(n = 0)	0	0	0	0	0	0	0
Shillong(n = 0)	0	0	0	0	0	0	0
Shimla(n = 5)	4(100.0)	4(100.0)	4(100.0)	0(0.0)	0(0.0)	4(100)	4(100)
Wardha(n = 2)	2(100)	2(100)	2(100)	0(0.0)	0(0.0)	2(100)	2(100)
Vellore(n = 0)	0	0	0	0	0	0	0
New Delhi(n = 10)	9(90.0)	9(90.0)	9(90.0)	1(10.0)	1(10.0)	10(100)	10(100)
Total (n = 18)	16(88.9)	16(88.9)	16(88.9)	1(5.6)	1(5.6)	18(100)	18(100)

Abbreviations: CHL (Chloramphenicol), Amox (Amoxicillin) COT (Co-trimoxazole), NA (Nalidixic acid), CIP (Ciprofloxacin), CFX (Cefixime), CTX (Ceftriaxone).

Zone diameters as per CLSI 2015: Susceptible/Resistant–CHL ≥18mm/≤17mm, AMOX ≥23mm/≤22mm, COT ≥16mm/≤16mm, CIP ≥31mm/≤30mm, CFX ≥19mm/≤18mm, CTX ≥23mm≤/22mm [14] (intermediate zone diameters are considered as resistant).

### Antimicrobial Susceptibility

Antimicrobial susceptibility to antityphoidal antibiotics is presented in Tables 1 and 2. All the isolates were susceptible to ceftriaxone and cefixime. Percentage of isolates susceptible to ciprofloxacin, chloramphenicol, amoxicillin and co-trimoxazole were 34.3, 99.1, 96.1 and 98.0 respectively. Table 3 shows the MIC values of ciprofloxacin and ceftriaxone for both serovars. There was complete concordance between susceptibility results of disk diffusion test and MIC interpretation. All nalidixic acid resistant (NAR) strains were also resistant to ciprofloxacin and all of the nalidixic acid susceptible (NAS) strains were susceptible to ciprofloxacin. There was no association between MLST sequence types and antimicrobial susceptibility.

**Table 3:** Minimum inhibitory concentration of *S*. Typhi and *S*. Paratyphi A for ciprofloxacin and ceftriaxone for all the isolates collected in the study period (n = 610).

Antimicrobial agent	MIC_50_(μg/ml)		MIC_90_ (μg/ml)		Median MIC (μg/ml)		(Max-Min) (μg/ml)	
	*S*.Typhi	*S*. Paratyphi A	*S*.Typhi	*S*. Paratyphi A	*S*.Typhi	*S*. Paratyphi A	*S*.Typhi	*S*.Paratyphi A
Ciprofloxacin	0.38	0.75	8	1	0.38	0.75	0.004->32	0.19–8
Ceftriaxone	0.016	0.094	0.047	0.19	0.016	0.094	0.019–0.75	0.023–0.75

CLSI 2015 MIC breakpoints Susceptible/Resistant: Ciprofloxacin ≤ 0.06/> 0.06 μg/ml, Ceftriaxone ≤ 1/> 1 μg/ml.

### MLST Analysis

All unique DNA sequences were submitted to genebank, the accession numbers of submitted sequences are given in Table 4. For phylogenetic relationships among *S*. Typhi isolates, forward and reverse DNA sequences were aligned and analyzed for each gene fragment. In multilocus sequence typing different sequences at each locus are assigned with specific allele numbers and each unique combination of alleles, often called as allelic profile is assigned a sequence type which is the unambiguous descriptor of the strain. The unique sequence types for *S*. Typhi and *S*. Paratyphi A are given in Table 5.

**Table 4:** Accession numbers of the unique sequences found in MLST of *S*. Typhi and *S*. Paratyphi A in India.

Gene	Allele No.	Accession No.	Gene	Allele No.	Accession No.
*aroC*	1	kT346341	*aroC*	45	KT346418
*hemD*	1	KT346343	*hemD*	8	KT364420
*hemD*	2	KT346344	*dnaN*	4	KT364419
*dnaN*	1	KT346342	*hisD*	44	KT364421
*hisD*	1	KT346345	*purE*	27	KT364422
*purE*	1	KT346346	*sucA*	9	KT364423
*sucA*	1	KT346347	*sucA*	56	KT364424
*thrA*	5	KT346348	*thrA*	8	KT364425
*thrA*	9	KT346349			

**Table 5:** Allelic configurations of all known *S*. Typhi and *S*. Paratyphi A sequence types available in online database [12].

Organism	Sequence Type	*aroC*	*dnaN*	*hemD*	*hisD*	*purE*	*sucA*	*thrA*	*eBURST Group*
*S*. Typhi*	**1**	**1**	**1**	**1**	**1**	**1**	**1**	**5**	**7**
	**2**	**1**	**1**	**2**	**1**	**1**	**1**	**5**	**7**
	**3**	**1**	**1**	**2**	**1**	**1**	**1**	**9**	**7**
	8	1	1	2	3	1	1	5	7
	890	1	1	2	353	1	1	5	7
	892	305	1	2	1	1	1	5	7
	911	1	1	2	183	1	1	5	7
	1856	1	1	340	1	1	1	5	7
	1919	1	1	1	1	1	1	9	7
*S*. Paratyphi A*	**85**	**45**	**4**	**8**	**44**	**27**	**9**	**8**	**10**
	**129**	**45**	**4**	**8**	**44**	**27**	**56**	**8**	**10**
	130	45	4	8	44	50	9	8	10
	479	164	4	8	181	27	9	8	10
	494	45	77	8	167	27	9	8	10
	495	45	8	8	127	27	9	8	10
	1618	45	4	8	44	140	9	8	10

* The sequence types found in our study are given in bold format.

All 251 *S*. Typhi strains subjected to MLST showed monophyletic lineage and clustered in to 3 Sequence Types—ST1, ST2 and ST3. There were 223 ST1, 27 ST2 and 1 ST3 sequence types found in our study in a period of three years. DNA sequence analysis for seven housekeeping genes involved in MLST showed that ST2 is the founder sequence type for *S*. Typhi from which ST1 and ST3 sequence types are evolved. ST1 and ST3 are the single locus variants of ST2 sequence type. ST1 has single point mutation in *hemD* gene having *hemD1* allelic type while ST2 and ST3 have *hemD2* allelic variant. In case of ST3 there is a single point mutation found in *thrA* gene. ST3 has *thrA9* allelic type and ST1 and ST2 have *thrA5* allelic type. *S*. Paratyphi A contained two sequence types ST85 and ST129. Out of total 18 *S*. Paratyphi A strains sequenced for MLST 14 were ST129 and 4 were ST85 (Table 6). ST129 is a single locus variant of ST85 in which *sucA* gene shows single point mutation and has *sucA56* while ST85 has *sucA8* allelic type. ClonalFrame analysis also revealed that the variations was due to single nucleotide polymorphism and no recombination event was seen.

**Table 6:** Distribution of sequence types for 7 housekeeping genes in strains selected from all center.

Center Name	*S*.Typhi				*S*. Paratyphi A		
	Total	Sequence Type 1	Sequence Type 2	Sequence Type 3	Total	Sequence Type 129	Sequence Type 85
Chennai	8	8	0	0	1	1	0
Orissa	15	15	0	0	0	0	0
Shillong	15	15	0	0	0	0	0
Shimla	24	24	0	0	5	5	0
Wardha	9	9	0	0	2	2	0
Vellore	90	83	8	1	0	0	0
New Delhi	90	72	19	0	10	6	4
Total	251	223	27	1	18	14	4

### Phylogenetic Analysis

**Fig 1:**
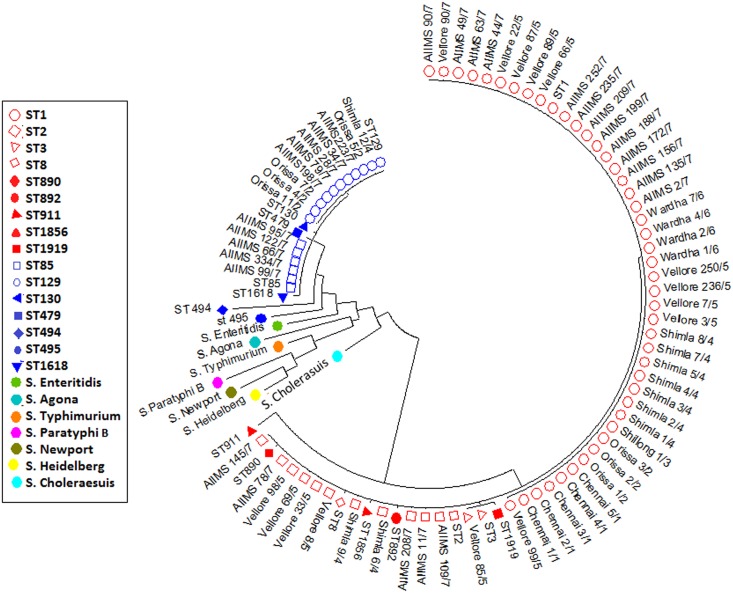
Phylogenetic tree constructed using Maximum Likelihood method and inferred with the Tamura-Nei model. *S*.Typhi sequence types are represented as red color shapes and *S*. Paratyphi A sequence types are represented as blue color shapes.

Maximum likelihood tree revealed three different clusters for *S*. Typhi and two clusters for *S*. Paratyphi A in our isolates. For the clarity of picture, only 20% of ST1 isolates are shown in phylogenetic tree. The tree with the highest log likelihood (-5988.0604) is shown as Fig 1.

**Fig 2:**
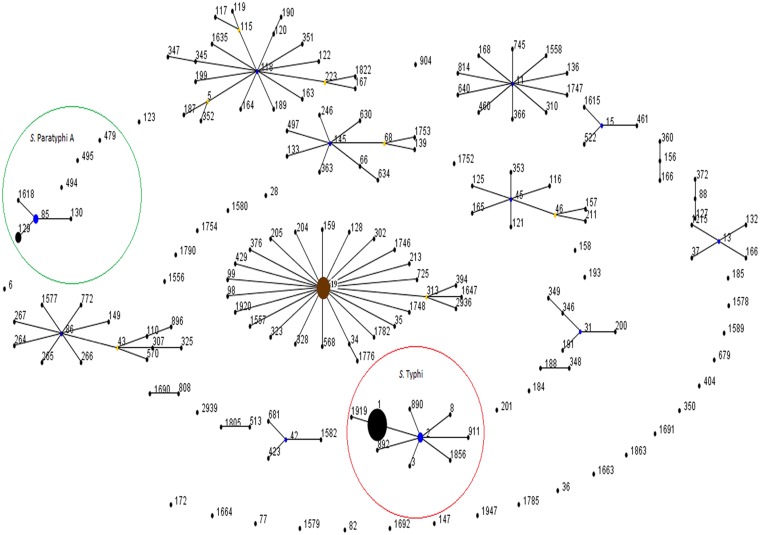
Population snapshot of *S*. Typhi and *S*. Paratyphi A and reference serovars. All sequence types are displayed as a single eBURST diagram. There are single allelic profiles representing each sequence type of different serotypes used for reference and all the allelic profiles for strains included in the study. Size of the dots corresponds to the number of iterations of that sequence type present in the data (ST1, ST2, ST3, ST85 and ST129 sequence types that were found in our study). Blue dots represent the group founder and yellow dots represent subgroup founder for each eBURST groups. Eburst analysis shows that on the basis of MLST sequences that *S*. Typhimurium sequence type ST19 is the founder strain for *S*. *enterica* family (Shown in brown colour). *S*. Typhi sequence types are highlighted in red Circle and belong to single group. Similarly *S*. Paratyphi Sequence types are highlighted in green circle, showing a group of four sequence type and three singleton sequences.

Data analysis using eBURST algorithm showed that *S*. Typhimurium is the founder sequence type for *S*.*enterica* in our dataset. Total 17 eBURST groups (eBG) were found and 34 sequence types were segregated as singletons in the sequence types of the 9 serotypes used for analysis. *S*. Typhi sequence types belonged to single eBURST group eBG 7 in our dataset (S1 Text). The founder sequence type for *S*. Typhi is ST2. Seven sequence types ST1, ST3, ST8, ST890, ST892, ST911 and ST1856 are single locus variants of ST2 while ST1919 is further evolved from ST1 and is double locus variant of ST2 (Fig 2). *S*. Paratyphi A sequence types formed an eBURST group eBG 10 having four sequence types ST85, ST129, ST130 and ST1618 where ST85 is the sequence founder for the group while three sequence types ST479, ST494 and ST495 were diversified as singletons (Fig 2).

A population snapshot of all sequence types available for *S*. Typhi, *S*. Paratyphi A and reference serovars i.e, *S*. Agona, *S*. Paratyphi A, *S*. Newport, *S*. Enteritidis, *S*. Typhimurium and *S*. Heidelberg is displayed as a single eBURST diagram by setting the group definition parameter to zero shared allele sothat all the sequence types were grouped in a single group.

### Geographical Distribution of Sequence Types

**Fig 3:**
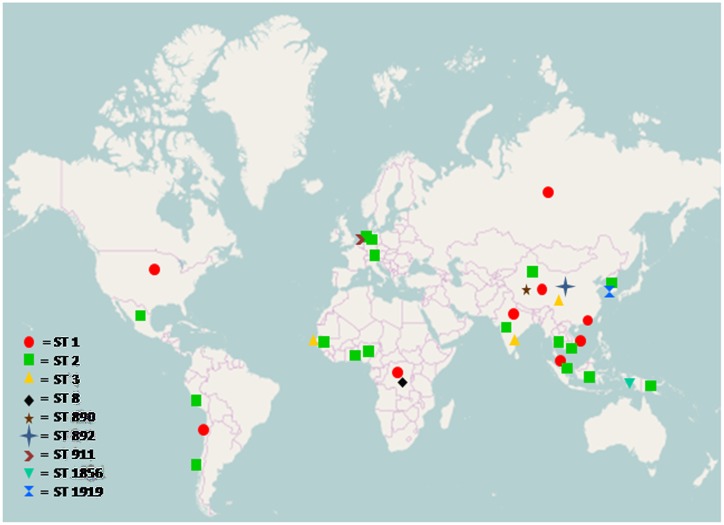
Geographical distribution of *Salmonella enteric*a serovar Typhi sequence types, analyzed from MLST dataset submitted to online database (http://mlst.warwick.ac.uk/mlst/dbs/Senterica). ST1: Hong Kong, Chile, India, Vietnam, China, Malaysia, Zaire, Russia, USA. ST2: Korea, China, Malaysia, Indonesia, India, Hong Kong, Papua New Guinea, Benin, Switzerland, Nigeria, Thailand, Vietnam, Germany, Netherland, Peru, Senegal, Mexico. ST3: China, India, Senegal. ST8: Zaire. ST890: China. ST892: China. ST911: Netherland. ST1856: Indonesia. ST1919: Korea. The map was retrieved from the website http://www.openstreetmap.org.

**Fig 4:**
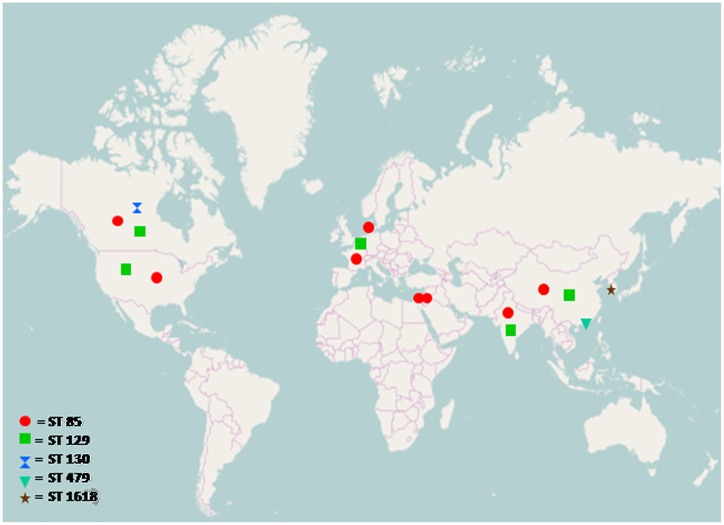
Geographical distribution of *Salmonella enteric*a serovar Paratyphi A sequence types analyzed from MLST dataset submitted to online database (http://mlst.warwick.ac.uk/mlst/dbs/Senterica). ST 85: France, China, Jordan, Israel, USA, Canada, India, Denmark. ST 129: China, Canada, India, Belgium, USA. ST 130: Canada. ST 479: Hong Kong. ST 1618: Korea *Geographical location of ST494 and ST495 is not reported in database**.** The map was retrieved fromthe website http://www.openstreetmap.org.

The prevalence of all sequence types of *S*. Typhi and *S*. Paratyphi A was analyzed from MLST database and plotted on a world map which shows the prevalence of *S*. Typhi serotype ST2 all over the world, while other sequence types are localized to specific regions only, similarly most prevalent *S*. Paratyphi A sequence type was ST85 as shown in Figs 3 and 4.

## Discussion

Enteric fever is a systemic disease which remains an important public health problem in India and other developing countries [27]. The emergence of antimicrobial resistance has raised therapeutic challenges and is responsible for an increase in morbidity and mortality. As the infection is no longer restricted by geographical boundaries, it is important to understand the distribution and spread of the causative organisms.

There are many typing approaches currently in use which enable typing of unrelated strains, with varying accuracy, discriminatory power, and reproducibility [9]. The choice of methods depends on the relevance and use of typing [8]. For monitoring short-term, local outbreaks molecular typing methods based on hyper-variable loci are appropriate but they do not reflect evolution over longer periods as can be detected by analysis of conserved genes [28,29].

MLST is one such method based on sequencing of housekeeping genes which provides an ideal balance between discriminatory power, reproducibility, reliability and portability. The MLST data is available in public domain and can be entered from decentralized sources and the allele designation for MLST can be readily extracted and compared within the database. MLST data thus can be readily compared among various laboratories and can be used to infer ancestral lineages by various clustering methods [30].

In this study seven centers were included from five states in different regions of India. India has 29 states, one National Capital Region and 7 union territories. As per the latest population consensus, each state and union territories contribute from 0.01 to 16% of total Indian population [31]. The present study represents the data from different corners of the country which make up about 21% of the total Indian population [31]. Therefore this data can be considered as representative to determine population structure of typhoidal salmonellae in India.

The present study found that predominating sequence types are ST1 and ST2 in *S*. Typhi while ST129 and ST85 predominates in *S*. Paratyphi A which suggest that typhoidal salmonellae are uniform in distribution and similar results were observed in previous studies and MLST database available in public domain [12,32].

In addition we found ST3, a rare sequence type of *S*. Typhi from southern India. Analysis of the public MLST *S*. *enterica* database revealed that at the time of writing only two other strains with ST3 sequence type have been reported from China and West Africa [12].

ST3 and ST1 are single locus variants of ST2 which is predicted to be founder strain of *S*. Typhi. In a study by C. Kidgell, ST1 and ST2 sequence types of *S*. Typhi were isolated from Eurasia, South America and Africa from 1918–1999 and 1981–2000 respectively, while from India only 3 strains were included at that time which belonged to ST2 sequence type isolated during 1984–1995 [11]. Many studies indicate that ST2 may be circulating internationally, while ST1 mainly persists in the Indian subcontinent as shown in previous studies [15,16].

*S*. Paratyphi A is an increasing cause of number of enteric fever cases in India and this is a concern because it is more resistant to antityphoidal antibiotics as compared to *S*. Typhi [33]. MLST analysis revealed two sequence types ST129 and ST85. MLST database analysis revealed that ST85 has been reported from France, China, Jordan, Israel, USA, Canada, India and Denmark while ST 129 has been reported China, Canada, India, Belgium and USA. Extensive search of MLST database showed that ST85 is the most prevalent strain worldwide. On the basis of MLST analysis of our dataset ST 129 is the most prevalent sequence type circulating in India and ST85 was reported from one center from north India.

The present information will help in understanding the evolution and spread of typhoidal salmonellae globally and contribute to the existing data of this major community acquired infection that is global public health concern. The findings of this study add knowledge to the global circulation of sequence types of *S*. *enterica*.

As ST3 has now been also identified in India and new sequence types are being added, there is a need to characterize a larger number of strains from different parts of world to understand the evolutionary history [34,35].

## Supporting Information

S1 Text eBURST analysis of *S*. *enterica* sequence types. (DOCX)
