# Supplementary material for: Typhoidal Salmonellae: Use of Multi-Locus Sequence Typing to Determine Population Structure
Source: PLoS One. 2016 Sep 12;11(9):e0162530. doi: 10.1371/journal.pone.0162530 (PMC5019401; doi:10.1371/journal.pone.0162530)
Supplement: S1 Text — (DOCX) [file pone.0162530.s001.docx]

eBURST Report - Mon Aug 29 18:03:02 IST 2016

No. re-samplings for bootstrapping = 1000

No. loci per isolate = 7 | No. identical loci for group def = 6 | No. groups = 17

Group 1: No. Isolates = 27 | No. STs = 27 | Predicted Founder = 19

Average ST Bootstrap

ST FREQ SLV DLV TLV SAT Distance Group Subgrp

19 1 22 4 0 0 1.15 100% 100%

313 1 8 17 1 0 1.73 28% 75%

429 1 6 17 3 0 1.88 0% 28%

376 1 6 17 3 0 1.88 1% 24%

205 1 6 17 3 0 1.88 1% 25%

204 1 6 17 3 0 1.88 2% 26%

159 1 6 17 3 0 1.88 3% 26%

128 1 6 17 3 0 1.88 6% 25%

302 1 5 20 1 0 1.84 0% 4%

1746 1 5 20 1 0 1.84 0% 4%

213 1 5 20 1 0 1.84 1% 4%

725 1 5 20 1 0 1.84 2% 4%

99 1 4 19 3 0 1.96 0% 7%

1748 1 4 19 3 0 1.96 0% 7%

35 1 4 19 3 0 1.96 0% 7%

1782 1 4 19 3 0 1.96 0% 8%

34 1 3 20 3 0 2.0 0% 0%

568 1 3 19 4 0 2.03 0% 0%

328 1 3 19 4 0 2.03 0% 0%

323 1 3 19 4 0 2.03 0% 0%

1557 1 2 21 3 0 2.03 0% 0%

394 1 2 7 16 1 2.61 0% 0%

1647 1 2 7 16 1 2.61 0% 0%

1920 1 1 23 2 0 2.03 0% 0%

98 1 1 21 4 0 2.11 0% 0%

2936 1 1 13 11 1 2.46 0% 0%

1776 1 1 6 16 3 2.80 0% 0%

Group 2: No. Isolates = 21 | No. STs = 21 | Predicted Founder = 118

Average ST Bootstrap

ST FREQ SLV DLV TLV SAT Distance Group Subgrp

118 1 12 8 0 0 1.4 99% 99%

115 1 5 12 3 0 1.9 18% 41%

223 1 5 11 4 0 1.95 14% 39%

345 1 4 13 3 0 1.95 5% 5%

120 1 4 12 4 0 2.0 3% 5%

5 1 4 11 5 0 2.05 8% 35%

122 1 4 10 6 0 2.1 4% 25%

1635 1 3 14 3 0 2.0 0% 0%

163 1 3 13 4 0 2.05 0% 0%

189 1 3 10 7 0 2.2 0% 0%

164 1 3 10 7 0 2.2 0% 0%

351 1 3 10 7 0 2.2 0% 0%

199 1 2 12 6 0 2.2 0% 0%

117 1 2 6 10 2 2.6 0% 0%

347 1 2 5 12 1 2.6 0% 0%

352 1 2 5 9 4 2.75 0% 0%

1822 1 1 9 8 2 2.55 0% 0%

119 1 1 9 8 2 2.55 0% 0%

190 1 1 8 9 2 2.6 0% 0%

167 1 1 7 8 4 2.75 0% 0%

187 1 1 3 11 5 3.0 0% 0%

Group 3: No. Isolates = 14 | No. STs = 14 | Predicted Founder = 86

Average ST Bootstrap

ST FREQ SLV DLV TLV SAT Distance Group Subgrp

86 1 8 4 1 0 1.46 71% 91%

43 1 7 6 0 0 1.46 61% 79%

1577 1 4 8 1 0 1.76 1% 0%

772 1 4 8 1 0 1.76 1% 0%

149 1 4 8 1 0 1.76 2% 0%

267 1 4 4 5 0 2.07 1% 11%

266 1 4 4 5 0 2.07 1% 11%

265 1 4 4 5 0 2.07 2% 11%

264 1 4 4 5 0 2.07 3% 11%

110 1 3 5 5 0 2.15 2% 1%

307 1 2 6 5 0 2.23 0% 0%

570 1 2 6 5 0 2.23 0% 0%

325 1 1 5 6 1 2.53 0% 0%

896 1 1 2 9 1 2.76 0% 0%

Group 4: No. Isolates = 11 | No. STs = 11 | Predicted Founder = 11

Average ST Bootstrap

ST FREQ SLV DLV TLV SAT Distance Group Subgrp

11 1 10 0 0 0 1.0 100% 100%

814 1 3 7 0 0 1.7 3% 0%

168 1 3 7 0 0 1.7 3% 0%

745 1 3 7 0 0 1.7 8% 0%

1558 1 2 8 0 0 1.8 0% 0%

136 1 2 8 0 0 1.8 0% 0%

1747 1 2 8 0 0 1.8 0% 0%

310 1 2 8 0 0 1.8 0% 0%

460 1 1 9 0 0 1.9 0% 0%

640 1 1 9 0 0 1.9 0% 0%

366 1 1 9 0 0 1.9 0% 0%

Group 5: No. Isolates = 11 | No. STs = 11 | Predicted Founder = 145

Average ST Bootstrap

ST FREQ SLV DLV TLV SAT Distance Group Subgrp

145 1 7 3 0 0 1.3 98% 96%

68 1 3 6 1 0 1.8 17% 21%

246 1 3 6 1 0 1.8 3% 0%

630 1 3 6 1 0 1.8 3% 0%

497 1 3 6 1 0 1.8 8% 0%

66 1 3 5 2 0 1.9 9% 2%

363 1 2 6 2 0 2.0 0% 0%

133 1 1 6 3 0 2.2 0% 0%

1753 1 1 5 4 0 2.3 0% 0%

634 1 1 5 3 1 2.4 0% 0%

139 1 1 2 6 1 2.7 0% 0%

Group 6: No. Isolates = 9 | No. STs = 9 | Predicted Founder = 45

Average ST Bootstrap

ST FREQ SLV DLV TLV SAT Distance Group Subgrp

45 1 6 2 0 0 1.25 88% 89%

46 1 4 4 0 0 1.5 38% 31%

353 1 2 6 0 0 1.75 0% 0%

165 1 2 4 2 0 2.0 0% 0%

125 1 2 4 2 0 2.0 0% 0%

121 1 2 4 2 0 2.0 0% 0%

116 1 2 4 2 0 2.0 0% 0%

211 1 1 3 4 0 2.37 0% 0%

157 1 1 3 4 0 2.37 0% 0%

Group 7: No. Isolates = 85 | No. STs = 9 | Predicted Founder = 2

Average ST Bootstrap

ST FREQ SLV DLV TLV SAT Distance Group Subgrp

2 6 7 1 0 0 1.12 98% 98%

1 72 3 5 0 0 1.62 17% 1%

890 1 3 4 1 0 1.75 4% 0%

8 1 3 4 1 0 1.75 4% 0%

911 1 3 4 1 0 1.75 6% 0%

3 1 2 6 0 0 1.75 0% 0%

1856 1 2 6 0 0 1.75 0% 0%

1919 1 2 2 4 0 2.25 3% 0%

892 1 1 6 1 0 2.0 0% 0%

Group 8: No. Isolates = 5 | No. STs = 5 | Predicted Founder = 13

Average ST Bootstrap

ST FREQ SLV DLV TLV SAT Distance Group Subgrp

13 1 4 0 0 0 1.0 88% 52%

1215 1 2 2 0 0 1.5 2% 0%

1328 1 2 2 0 0 1.5 4% 0%

1668 1 1 3 0 0 1.75 0% 0%

37 1 1 3 0 0 1.75 0% 0%

Group 9: No. Isolates = 5 | No. STs = 5 | Predicted Founder = 31

Average ST Bootstrap

ST FREQ SLV DLV TLV SAT Distance Group Subgrp

31 1 3 1 0 0 1.25 58% 16%

346 1 2 2 0 0 1.5 21% 0%

191 1 1 2 1 0 2.0 0% 0%

200 1 1 2 1 0 2.0 0% 0%

349 1 1 1 2 0 2.25 0% 0%

Group 10: No. Isolates = 14 | No. STs = 4 | Predicted Founder = 85

Average ST Bootstrap

ST FREQ SLV DLV TLV SAT Distance Group Subgrp

85 5 3 0 0 0 1.0 61% 13%

1618 1 2 1 0 0 1.33 4% 0%

130 1 2 1 0 0 1.33 10% 0%

129 7 1 2 0 0 1.66 0% 0%

Group 11: No. Isolates = 4 | No. STs = 4 | Predicted Founder = 15

Average ST Bootstrap

ST FREQ SLV DLV TLV SAT Distance Group Subgrp

15 1 3 0 0 0 1.0 74% 14%

1615 1 1 2 0 0 1.66 0% 0%

461 1 1 2 0 0 1.66 0% 0%

522 1 1 2 0 0 1.66 0% 0%

Group 12: No. Isolates = 4 | No. STs = 4 | Predicted Founder = 42

Average ST Bootstrap

ST FREQ SLV DLV TLV SAT Distance Group Subgrp

42 1 3 0 0 0 1.0 60% 12%

681 1 2 1 0 0 1.33 4% 0%

1582 1 2 1 0 0 1.33 10% 0%

423 1 1 2 0 0 1.66 0% 0%

Group 13: No. Isolates = 3 | No. STs = 3 | Predicted Founder = 88

Average ST Bootstrap

ST FREQ SLV DLV TLV SAT Distance Group Subgrp

88 1 2 0 0 0 1.0 29% 0%

372 1 1 1 0 0 1.5 0% 0%

127 1 1 1 0 0 1.5 0% 0%

Group 14: No. Isolates = 3 | No. STs = 3 | Predicted Founder = 156

Average ST Bootstrap

ST FREQ SLV DLV TLV SAT Distance Group Subgrp

156 1 2 0 0 0 1.0 35% 0%

360 1 1 1 0 0 1.5 0% 0%

166 1 1 1 0 0 1.5 0% 0%

Group 15: No. Isolates = 2 | No. STs = 2 | Predicted Founder = None

ST FREQ SLV DLV TLV SAT Distance

1805 1 1 0 0 0 1.0

513 1 1 0 0 0 1.0

Group 16: No. Isolates = 2 | No. STs = 2 | Predicted Founder = None

ST FREQ SLV DLV TLV SAT Distance

188 1 1 0 0 0 1.0

348 1 1 0 0 0 1.0

Group 17: No. Isolates = 2 | No. STs = 2 | Predicted Founder = None

ST FREQ SLV DLV TLV SAT Distance

1690 1 1 0 0 0 1.0

808 1 1 0 0 0 1.0

Singletons: size 34

1579

1578

1754

1752

1556

1947

193

82

1692

185

1691

184

77

172

404

201

2939

1863

158

1664

350

1663

147

36

495

494

28

6

123

1790

679

479

1785

1589
